# Distinct plasma chemokines and cytokines signatures in *Leishmania guyanensis*-infected patients with cutaneous leishmaniasis

**DOI:** 10.3389/fimmu.2022.974051

**Published:** 2022-08-25

**Authors:** Tirza Gabrielle Ramos de Mesquita, José do Espírito Santo Junior, Luan Diego Oliveira da Silva, George Allan Villarouco Silva, Felipe Jules de Araújo, Suzana Kanawati Pinheiro, Herllon Karllos Athaydes Kerr, Lener Santos da Silva, Luciane Macedo de Souza, Samir Assad de Almeida, Krys Layane Guimarães Duarte Queiroz, Josué Lacerda de Souza, Cilana Chagas da Silva, Héctor David Graterol Sequera, Mara Lúcia Gomes de Souza, Anderson Nogueira Barbosa, Gemilson Soares Pontes, Marcus Vinitius de Farias Guerra, Rajendranath Ramasawmy

**Affiliations:** ^1^ Programa de Pós-Graduação em Medicina Tropical, Universidade do Estado do Amazonas, Manaus, Brazil; ^2^ Department of Molecular Biology, Fundação de Medicina Tropical Doutor Heitor Vieira Dourado, Manaus, Brazil; ^3^ Programa de Pós-Graduação em Imunologia Básica e Aplicada, Instituto de Ciências Biológicas, Universidade Federal do Amazonas, Manaus, Amazonas, Brazil; ^4^ Faculdade de Medicina Nilton Lins, Universidade Nilton Lins, Manaus, Brazil; ^5^ Department of Virology, Instituto Nacional de Pesquisas da Amazônia, Manaus, Brazil; ^6^ Genomic Health Surveillance Network: Optimization of Assistance and Research in The State of Amazonas – REGESAM, Manaus, Amazonas, Brazil

**Keywords:** *Leishmania guyanensis*, cytokines, chemokines, cutaneous leishmaniasis, growth factors

## Abstract

The immunopathology associated with Leishmaniasis is a consequence of inflammation. Upon infection with *Leishmania*, the type of host-immune response is determinant for the clinical manifestations that can lead to either self-healing or chronic disease. Multiple pathways may determine disease severity. A comparison of systemic immune profiles in patients with cutaneous leishmaniasis caused by *L. guyanensis* and healthy individuals with the same socio-epidemiological characteristics coming from the same endemic areas as the patients is performed to identify particular immune profile and pathways associated with the progression of disease development. Twenty-seven plasma soluble circulating factors were evaluated between the groups by univariate and multivariate analysis. The following biomarkers pairs IL-17/IL-9 (ρ=0,829), IL-17/IL-12 (ρ=0,786), IL-6/IL-1ra (ρ=0,785), IL-6/IL-12 (ρ=0,780), IL-1β/G-CSF (ρ=0,758) and IL-17/MIP-1β (ρ=0,754) showed the highest correlation mean among the patient while only INF-γ/IL-4 (ρ=0.740), 17/MIP-1β (ρ=0,712) and IL-17/IL-9 (ρ=0,707) exhibited positive correlation among the control group. The cytokine IL-17 and IL1β presented the greater number of positive pair correlation among the patients. The linear combinations of biomarkers displayed IP-10, IL-2 and RANTES as the variables with the higher discriminatory activity in the patient group compared to PDGF, IL-1ra and eotaxin among the control subjects. IP-10, IL-2, IL-1β, RANTES and IL-17 seem to be predictive value of progression to the development of disease among the *Lg*-infected individuals.

## Introduction

Leishmaniasis, a vector-borne infectious disease caused by *Leishmania spp* (*L.*), continues to be a public health burden in over 98 countries worldwide and still is a neglected tropical disease (1). *L.*-infected individuals can manifest a wide spectrum of clinical symptoms that is guided by the immunological status and the genetic background of the individual, the *L. spp*, and the environment. The disease outcome may range from asymptomatic, localized cutaneous lesions (cutaneous leishmaniasis (CL), severe mucosal lesions (mucosal Leishmaniasis (ML) and to life threatening visceral leishmaniasis (VL). CL and ML are also known as American Tegumentary leishmaniasis (ATL) in the American continent.

The major species that cause ATL in Brazil are *L. braziliensis (Lb)*, *L. guyanensis (Lg)*, *L. lainsoni*, *L. amazonensis*, *L. shawi*, *L. naiffi* and *L. lindenbergi*. *L. braziliensis* is responsible for the majority of the cases (2). However, in the Amazonas, *L. guyanensis* is the main etiological agent of ATL and represent 95% of the CL cases (3).

Different pathophysiological mechanisms are suggested to lead to the development of the different clinical manifestations of leishmaniasis and still, treatment with antimonials targeting the parasite continues to be the drug of choice with no headway in the development of new therapies (4). Pentavalent antimony (Sbv) is still the current first-line treatment in Brazil for CL and is administered daily by intravenous injection for 20 days. Patients often complain about the toxic side effects of the drug, and often need a second or third round of therapy when the drug fails to resolve disease (5). Treatment failure with Sbv in patients with CL can be as high as 45% (6, 7). Amphotericin B, pentamidine or miltefosine are alternative treatment options in patients with no response to Sbv therapy or relapsed.

High rate of treatment failure and the adverse effects of antimony as well as abandon of treatment require the search of new alternative treatment or therapy in combination with the current drug that may shorten the therapy in terms of days and lower toxic adverse effects. Furthermore, in endemic areas of leishmaniasis, only a proportion of individuals progress to disease development upon infection.

To understand the immunopathogenesis of CL caused by *L. guyanensis* (*Lg*-CL), this study attempted to find immunological pathways that could be involved in the progression to disease development in susceptible individuals and thus lead to the identification of targets for host-directed immunotherapies.

## Materials and methods

### Ethics approval and informed consent

This study was approved by the Research Ethics Committee of the Fundação de Medicina Tropical Doutor Heitor Vieira Dourado (FMT-HVD) and granted under the file number CAAE 09995212.0.0000.0005. All the participants or their responsible party for individuals less than 18 years old in the study provided written inform consent. Prior to the signing of the inform consent, explanation about the study was given to the participants and they were free to participate.

### Study population

This study was conducted in the peri-rural areas of Manaus, capital of the state of Amazonas, Brazil. The patients with active CL infected with *L. guyanensis (Lg-*CL*)* were followed at the FMT-HVD, a referral hospital for treating leishmaniasis patients. The control group comprises healthy controls (HC) living in the same endemic area as the patients, sharing similar environments.

### Identification of *Leishmania* spp

DNA was prepared from lesion biopsy specimens of all the participants with CL. The identification of the *Leishmania* species was performed by polymerase chain reaction (PCR) restriction fragment length polymorphism and direct nucleotide sequencing as described elsewhere (8). Only patients infected with *L. guyanensis* were included in the study.

### Cytokine assay by Luminex

5 mL of blood from patients with *Lg*-CL before antimonial treatment and from healthy controls were collected. Plasma was separated and kept frozen at -80°C until plasma cytokines assay.

The levels of FGF basic, Eotaxin, G-CSF, GM-CSF, IFNγ, IL-1β, IL-1RA, IL-2, IL-4, IL-5, IL-6, IL-7, IL-8, IL-9, IL-10, IL-12 (p70), IL-13, IL-15, IL-17A, IP-10, MCP-1, MIP-1α, MIP-1β, PDGF-BB, RANTES, TNFα and VEGF were determined using the multiplex cytokine commercial kit Bio-PlexPro-Human Cytokine GrpI Panel 27-Plex (Bio-Rad) according to the manufacturer’s instructions in the Bio-Plex 200 Protein Array System (Luminex Corporation).

### Statistical analysis

Plasma levels of chemokines and cytokines from patients and control groups were compared using Kruskal-Wallis test and the graph plots were constructed using R boxblot package version 3.5.1. The interrelatedness between all biomarker was assessed using Spearman correlation test with R corrplot package version 3.5.1. Values greater or equal to 0.7 were considered positive correlation, whereas values less than or equal to -0,7 were considered negative correlation. A color map matrix and a correlation network diagram (Cytoscape version 3.8.2) were built to represent the correlations between the plasma biomarkers analyzed.

Possible different biomarkers profile between patients and control groups were evaluated through Principal Component Analysis (PCA) clustering. PCA plot were generated based on the absolute abundance of biomarkers using R FactorMineR package version 3.5.1. The regions with 95% confidence levels were indicated with colored ellipses. A Linear Discriminant Analysis (LDA) were performed to determine which biomarker contributes most significantly to the discrimination of the study groups. Kruskal-Wallis (for classes) and pairwise Wilcoxon (for subclasses) tests were used to verify if the data were differentially distributed between the study groups. Logarithmic LDA score for discriminative features was built using a threshold of 2.0. The analysis was done through HutLab galaxy online platform.

Heatmap was generated through hierarchical agglomerative cluster analysis to estimate the pattern of biomarker expression in the study groups. The greatest appropriate distance metric and cluster linkage were defined based on Euclidean distance and complete linkage methods, respectively. Heatmap was created using gplots package in R version 3.5.1.

## Results

### Characterization of the study population

This study has a cross-sectional design and consisted of 354 patients with *Lg-*CL (90 males and 264 females) and 376 (107 males and 269 females) healthy individuals with the same socio-epidemiological situations coming from the same endemicity area as the patients. The average age of the male patients with *Lg-*CL and male healthy controls were 39.8 ± 1.57 and 45.2 ± 1.58 years old, respectively. Age of female patient with *Lg-*CL and female healthy controls were34.6 ± 0.80 and 43.7 ± 1.80 years old, respectively. The skin lesions of patients were mostly located in the upper and lower limbs. All patients presented recent lesions, ranging three weeks to five weeks. The healthy individuals have no history of leishmaniasis and most of them are agriculture workers as the patients. The study population was devoid of HIV and had no history of diabetes mellitus, cardiac, renal and hepatic disease. The patients had fewer or equal to six lesions and treatment-naïve at the time of enrolment. Most of the patients had only one lesion. All the patients were first time infected. The exclusion criteria were patients with *Lg*-CL with previous history of leishmaniasis. Pregnant women were excluded.

### *Leishmania* infection triggers intense pro-inflammatory immune response characterized by elevated plasma levels of chemokines, cytokines and growth factors

Plasma circulating levels of 27 biomarkers were assessed in *Leishmania*-infected patients and healthy individuals from control group. Prior to comparison of the plasma biomarkers between patients with CL and healthy control, biomarkers were investigated if age and sex influenced the plasma levels by linear correlations using linear regressions of the R package (**Supplementary Figures 1A–D**). The levels of biomarkers were not influenced by sex. However, the levels of IFN-γ, IL-1β, IL12p70, IL-6, IL-17, TNFα, IL-4, IL-5, IL-13, CXCL8, CXCL10, CCL4, CCL5, GM-CSF, and PDGF were influenced by age. The P value of these biomarkers were adjusted for age using the general linear model of R package.

Patients with *Lg*-CL showed increased levels of IL-1β, IL-6, IL-7, TNF-α, IL-12, IFN-γ, IL-17, IL-1Ra, IL-2, IL-15, IL-4, IL-9 and IL-10 compared to the healthy individuals (**Supplementary Figure 2**). Likewise, higher levels of FGF-basic, PDGF, VEGF, G-CSF and GM-CSF growth factors were found in the patients group. The plasma levels of MCP-1, IL-13 and IL-5 were similar among the individuals from patient and control groups (p<0.05). These findings demonstrate that the *Leishmania* infection drives a broader and stronger polyclonal T cell response with the engagement of different leukocyte populations.

*Leishmania* induced high expression of MIP-1α, eotaxin, MIP1-β, RANTES, IP-10 and IL-8. These chemokines are crucial for the chemoattraction of natural killer cells (MIP1-β), neutrophils (MIP-1α and IL-8), eosinophils (eotaxin), T cells and dendritic cells (RANTES). The IP-10 was the biomarker with the highest level observed in the patients. The leading role of IP-10 is the activation and maintenance of immune response by chemoattraction of monocytes, T cells, NK and dendritic cells; and the promotion of T cell adhesion to endothelial cells.

### Several cytokines, chemokines and growth factors are highly correlated in *Leishmania*-infected patients

**Figure 1:**
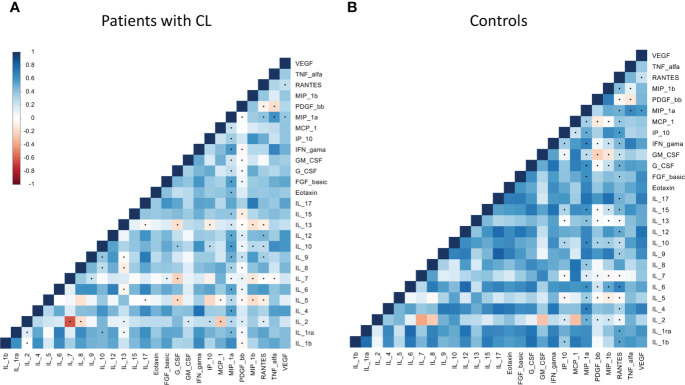
Correlation matrices within chemokines, cytokines and growth factors among patients with cutaneous leishmaniasis **(A)** and healthy controls **(B)** groups. Increasing values are represented by colors codes: blue for positive correlation and red for negative correlation. Significance: p< 0.05.

The correlation analysis demonstrated a strong positive association among many biomarkers in patients with CL (**Figures 1A, B**). Total mean correlation (ρ) of 0.41 and 0.31 were observed among patients with CL and control group, respectively.

From a total of 340 biomarkers pairs, a mean of 30 pairs showed positive correlation in the patient group (**Supplementary Table 1A**). The biomarkers pairs that showed the highest correlation mean in the patient group were: Among the patients with *Lg*-CL, strong positive correlations of IL17/IL12, IL17/IL4, IL17/IL9, IL17/MIP1α, IL17/MIP1β, IL1RA/IL1β, IL6/IL1β, IL15/IL1β, G-CSF/IL1β, IFNγ/IL1β, MCP-1/IL1β, IL6/IL1RA, IL12/IL1RA, G-CSF/IL1RA, IL9/IL4, IFNγ/IL4, Eotaxin/IL4, IL8/IL6, IL12/IL6, G-CSF/IL6, MCP1/IL8, MIP1α/IL9, MIP1β/IL9, FGF-B/IL12, VEGF/IL12, IFNγ/IL15, IFNγ/G-GSF-F, and GM-CSF/MCP1. Only IFNγ/IL4, IL17/MIP1β and IL17/IL9 showed positive correlations in the healthy controls group (**Supplementary Table 1B**). The cytokine IL-17 and IL-6 presented the greater number of positive pairs correlation (**Supplementary Table 1A**), which may indicate their key role in the immune response against the *Leishmania*-infection.

**Figure 2:**
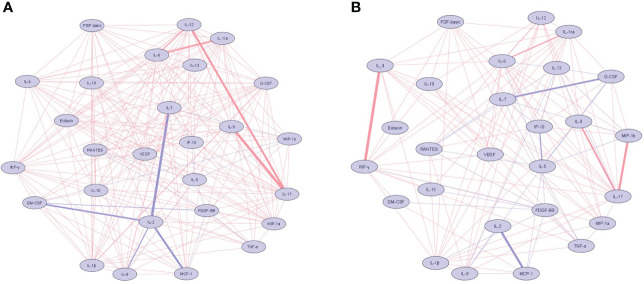
Network of cytokine correlations in the patients with cutaneous leishmaniasis **(A)** and healthy controls **(B)** groups. Nodes symbolize cytokines and connecting lines represent a Spearman’s correlation between two biomarkers. Positive correlations are indicated by red lines, while blue lines represent negative correlations. The absolute value of the correlation is represented by the width of the lines. The thickness and saturation of the lines were proportional to the strength of correlation. Biomarkers were arranged based on the number of connections from minimum to maximum. The network diagram was constructed using Cytoscape software version 3.8.2.

Correlations network based on Spearman correlations of the 27 plasma biomarkers are shown among the patients with *Lg*-CL in **Figure 2A** and the healthy controls in **Figure 2B**. Positive and negative correlations are shown in red and blue, respectively. The thickness of the connections is proportional to the Spearman rank coefficient rho value. IL-17 and IL-1β were the most relevant markers revealing positive relationships among the patients with *Lg*-CL. IL-2 showed the highest negative correlations among the patients with *Lg*-CL. Among the healthy controls, strong positive correlations were observed between INF-γ and IL-4 whereas IL-7 showed strong negative relationship with G-CSF.

### High discriminatory activity of plasma biomarkers may predict disease clinical outcome

**Figure 3:**
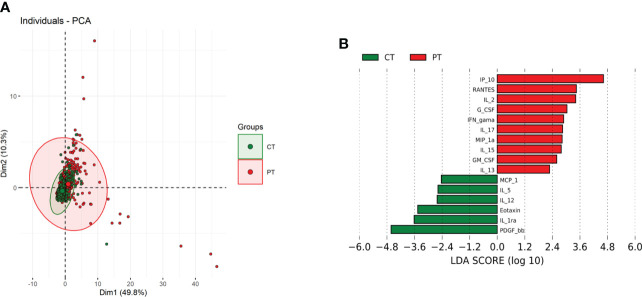
*Principal component analysis (PCA) and Linear discriminant analysis (LDA) of reciprocally expressed biomarkers*. **(A)** Scatterplot of the first two principal components (Dim1 and Dim2) showing two main groups, comprising 60.1% of total variance. Biomarkers from patients with cutaneous leishmaniasis (red closed circles) and of healthy control group (green closed circles) clustered in two different groups with overlapping regions. Colored ellipses indicate regions with 95% confidence levels. **(B)**. Separation of biomarkers within two studied groups based on its discrimination power.

Correlation pattern of cytokines expressions were assessed in the whole multivariate set of cytokines and chemokines in the study groups. Principal component analysis did not completely segregate the patients with *Lg*-CL and control groups into separate clusters (**Figure 3A**). However, patients with *Lg*-CL were spread over a wide region, which denotes a higher variance of biomarker levels in patients than in controls.

Additionally, the linear combinations of biomarkers demonstrated that IP-10, IL-2 and RANTES are the variables with the higher discriminatory activity in the patient group, whereas PDGF, IL-1RA and eotaxin (CCL11) seemed to be more relevant among the control subjects (**Figure 3B**). This correlation may indicate a functional association between these biomarkers and the immunopathology of leishmaniasis. In other words, IP-10, IL-2 and RANTES could be a predictive value of clinical parameters for progressing to disease, while PDGF, IL-1Ra and eotaxin may be protective biomarkers.

### Cluster analysis of biomarkers within the patient and control groups show diffuse expression pattern

**Figure 4:**
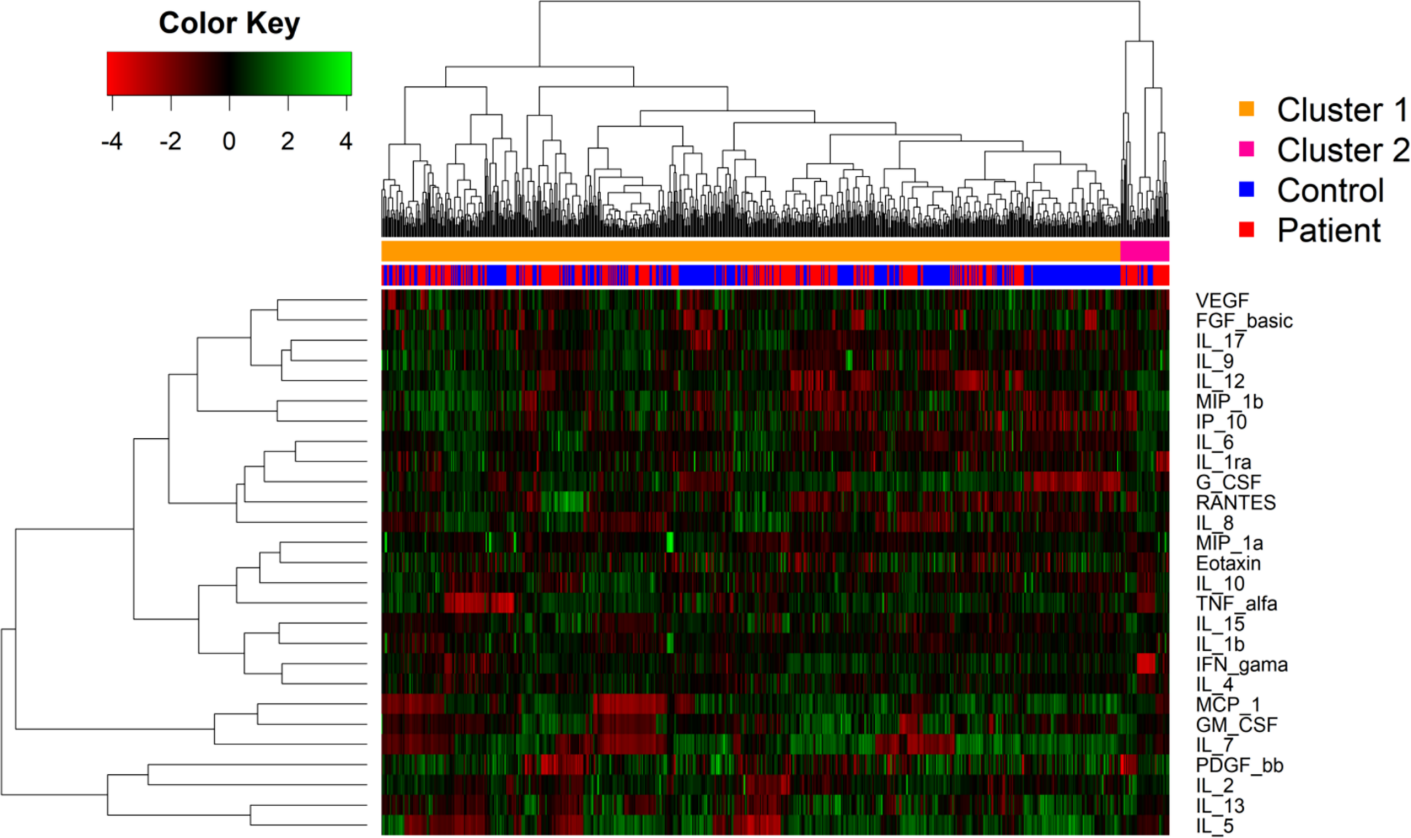
*Heat map showing relative expression of biomarkers levels patients with cutaneous leishmaniasis and in healthy controls.* Biomarker levels are expressed by color codes. Green represents the lowest and red the highest cytokine levels in the color key on the left. The rows denote the cytokines and the columns the infection status. The cluster analysis demonstrates the level of correlation among the variables represented by dendrograms. The distance between the two clusters is represented by the height where the two clusters merge.

We next addressed how the global cytokines and chemokines expression would cluster in a heatmap, without considering the patient clinical manifestations. The sample dendrogram showed two main clusters. However, hierarchical clustering analysis revealed that the patients did not completely segregate from the control group based on their biomarker expression (**Figure 4**).

The expression of biomarkers displayed a diffusion pattern. This may have occurred because of underlying unknown inflammatory conditions in some individuals of the control group, which also may increase the levels of the biomarkers analyzed. Moreover, the cluster 2 was predominantly composed by patients with the IFN-γ showing the highest levels. Additionally, as observed in the linear discriminant analysis, the levels of RANTES, IP-10 and IL-2 were higher among patients with *Lg*-CL compared to the healthy individuals, which confirms the possible role of these cytokines in the immunopathogenesis of leishmaniasis.

## Discussion

Currently, there is no vaccine for leishmaniasis and pharmacotherapy with SbV is often ineffective with toxic side effects. Upon infection with the parasite, a T_H_-1 type immune response is required to control the pathogen and cure the disease. An immune response involves a well-coordinated interplay of antigen-recognizing cells and signaling molecules to mount a sufficient specific response against the pathogen without provoking any tissue damage. Patterns of circulating cytokines and chemokines in patients with *Lg*-CL may provide useful information to the understanding of the underlying mechanisms of leishmaniasis to enhance the search of novel therapies such as immunotherapy in combination to conventional therapy.

High plasma levels of inflammatory cytokines IL-1β, IL-12, IFN-γ, TNF-α, IL-6 and IL-17 were observed in patients with *Lg*-CL compared to healthy controls. Hierarchical analysis showed a strong link between IL-1β and IL-6. IL-12 is released from dendritic cells, CD4 and CD8 T lymphocytes. Notably, IL-1β, IL6 and TNF-α are mainly derived from innate cells. Altogether, this may suggest a strong activation of innate cells among the patients with CL culminating in the release of proinflammatory cytokines. Of note, it is well known in animal models that resistance to *Leishmania-* infection is driven by a T_H_-1 immune response (9). Exacerbated inflammatory processes and non-specific cytotoxic response participate in the pathogenesis of *Lb*-CL (10–13). Furthermore, high expression of cytotoxic and inflammatory-related genes was observed in biopsy specimens of skin lesion from patients with CL caused by *L. braziliensis* (*Lb*-CL) (14–17). Recently, a study reported high expression of senescence associated secretory phenotype related genes, CCL3, CCL8, CXCL1, CXCL11, CXCL13, CXCL8/IL8 (associated with the recruitment of inflammatory immune cells) and IL-6, IL-15 and IL-1β (inflammatory cytokines) in patients with *Lb*-CL. IFN-γ, TNF-α, CXL10, and CCL4 also were highly expressed (18). Circulating senescent T cells, with high inflammatory profile, were linked to systemic inflammation and lesion size in patients with *Lb*-CL (19). Transcriptome analysis from skin lesion of patients with *Lb*-CL showed strong signature of inflammasome activation and release of IL-1β, IFN-γ and TNF-α (11, 12). This study observed increased plasma levels of CCL3, CXCL8, IL6, IL-15, IL-1β, IFN-γ and TNF-α among the patients with *Lg*-CL compared to the healthy controls.

High expression levels of IL-10, TGF-β, TNF-α, IFN-γ, IL-12B, CCL2/MCP1, CCL3/MIP1α, CCL5/RANTES and CXCL10/IP10 (Inflammatory profiles) were observed in early lesions of patients with *Lb*-CL (20). In this study high plasma levels of inflammatory cytokines, IFN-γ, TNF-α, IL-12 and chemokines CCL2/MCP1, CCL3/MIP1α, CCL5/RANTES and CXCL10/IP10 were observed in patients with *Lg*-CL compared to healthy controls. All of the patients with *Lg*-CL participating in this study had recent lesions. Control of *L.*-infection is mediated by T lymphocytes upon early induction of IL-12 that leads to a T_H-_1 mediated immune response releasing IFN-γ to activate macrophages to keep the parasite in check (21). CXCL10 is cited to protect against *L. major* infection in mice by stimulating NK cell cytotoxic activity (22). IFN-γ and CXCL10 are reported to activate T lymphocytes and development of a Th1 response during active VL (23). Some studies cited that NK cells are protective against leishmaniasis while others suggested the contrary indicating a cytotoxic role contributing to tissue damage in *Lb*-infection (24–26). A recent study observed increased frequency of cytotoxic NK cells in cell cultures of PBMC stimulated with *Lb*-antigens from patients with *Lb*-CL before treatment, while high frequencies of exhaustion NK cells during treatment (27). Administration of Met-RANTES (an anti-CCL5) in C57/BL6 mice render them susceptible to *L. major* (28). Immunohistochemistry of biopsy specimens of lesions from patients with *Lb*-CL or mice showed the presence of T cells infiltration, macrophages, B cells, NK cells and granulocytes (29, 30).

Increased plasma levels of IL-4, IL-9 and IL-10 were observed in patients with CL while levels of IL-5 and IL-13 were similar among both groups. IL-4 and IL-10 are known to modulate T_H_1-mediated immune response (31). The increased levels of these cytokines in patients with *Lg*-CL may suggest that the patients react to counter the observed inflammatory process.

Increased levels of FGF-basic, PDGF, VEGF, G-CSF and GM-CSF growth factors were observed among the patients with CL. Growth factors modulate inflammatory process in chronic diseases and may indicate the need of tissue repair. VEGF was suggested to be important in the resolution of leishmaniasis lesions (32). GM-CSF is important during the inflammatory stage of wound healing to induce the migration of neutrophils (33). GM-CSF also induces the migration and proliferation of endothelial cells (34) and upregulate IL-6 (35). Patients with *Lb*-CL exhibit elevated levels of circulating Th1 lymphocytes, cytokines and chemokines (36). GM-CSF is reported to promote protection against *Leishmania-*infection (37).

The increased levels of chemokines among the patients with *Lg*-CL may suggest that the patients are reacting to induce wound healing. Chemokines induced the migration of several proinflammatory cells type to the site of lesions (38), contributing to wound healing by tissue remodeling and angiogenesis (39). IP-10 and MIP-1α attract monocytes, macrophages, and activated T cells to sites of infection to enhance wound healing and parasite elimination (40, 41). However, overexpression of IP-10 may result in delay of wound healing by enhancing a potent inflammatory response through the recruitment of lymphocytes (42). During *Leishmania*-infection, the chemokines MIP1-β and eotaxin are released by monocytes and can lead to an intense inflammatory response (43).

In this study, patients with *Lg*-CL displayed a distinct expression profile of proinflammatory cytokines compared to the healthy controls group. IL-1β displayed higher number of positive correlations among the patients with *Lg*-CL. Several studies have shown that tissue destruction initiated by cytolytic CD8^+^ T cells trigger NLRP3 inflammasome activation leading to the secretion of IL-1β (10, 11, 14, 44–47). IL-1β is a proinflammatory cytokine involved in the CL pathogenesis (48). Blocking components of NLRP3 or IL-1β decreased the severity of the disease in animal models (11). Higher frequencies of CD8^+^ T cells have been observed in skin lesions of patients with *Lb*-CL and are unrelated to parasite load, suggesting CD8^+^ T cells contribute to the immunopathogenesis of CL (46, 47). CD8^+^ T cells cocultured with *Lb*-infected macrophages enhanced the expression of NLRP3, AIM2, and CASP1/5 culminating to the release of IL-1β. High expression of NLRP3, AIM2, and CASP1/5 have also been observed in skin lesions of patients with *Lb*-CL (49).

The immune response in infectious diseases to control the pathogen depends on the type and magnitude of the response. An excess of proinflammatory cytokines may lead to tissue destruction. RNA-seq of skin biopsies from patients with *Lb*-CL highlights an increase of inflammatory transcript of *IFNG* and *TNFA* (16). A recent study showed that a transcriptional signature including IL-1β may predict clinical outcomes of *Lb*-infection (15). Notably, IL-1β and IFN-γ were suggested to predict clinical outcome in patients with *Lb*-CL (15). Interestingly, variants in the *IL1B* and *IFNG* genes are associated with susceptibility to *L.*-infection (8, 50–52).

IL-17 showed positive correlations with IL-12, IL-9, MIP1α, MIP-1β and IL-4. Th17 cells and the IL-17/IL-23 axis have crucial role in immune-mediated inflammatory diseases (53). IL-17 induces fibroblasts, macrophages, endothelial and epithelial cells to release TNF-α, IL-6, NOS and metalloproteases to increase inflammation (54, 55). Plasma IL-6, IL-1β, IFNγ, TNFα and IL-17 were higher in patients with CL. Indeed, IL6 and IL-1β were positively correlated in CL patients in this study. Interestingly, TGF-β together with IL-6 or IL-1β downregulate FOXP3 and trigger the activation of RORgt, the transcription factor of IL17, and T_H_17 cells differentiation (56). TNFα and IL-1β induce the upregulation of IL-6 expression (57). High levels of IL-17 were correlated with the magnitude of cellular-infiltrates in *Lb*-CL (58). In biopsy specimens of skin lesion of patients with CL, IL-17, RORgt and IL-23 were observed (59–62). In light of all these studies and our findings, IL-17 seems to contribute to the pathogenesis and inflammatory processes of leishmaniasis.

A recent study observed that patients with *Lb*-CL who failed treatment after 60 days with Sb^v^ displayed lower plasma levels of eotaxin and IL-12p70 but increased G-CSF compared to successfully treated patients (63). Interestingly, PDGF, Eotaxin and IL1-RA showed the highest discriminatory activity among the healthy controls compared to the patients with *Lg*-CL in this study.

Blood transcriptional analysis of patients with active VL caused by *L. infantum (chagasi)*, patients under remission, asymptomatic and uninfected individuals revealed molecular pathways of activation of T lymphocytes *via* MHC class I and type I interferon signaling besides downregulation of pathways related to myeloid cells (monocytes and neutrophils) in patients with active VL, while patients under remission showed genes correlated with activation of Notch signaling pathway and increased proportions of B cells besides T lymphocytes *via* MHC class I and type I interferon signaling activation as patients with active VL (64). Patients under remission also displayed a negative regulation of IL-10 signaling pathway in the transcriptional profiles. Biopsy specimens of lesions from patients with *Lb*-CL also displayed a transcriptional profiling of positive type I interferon signaling (14). Human myeloid derived dendritic cells infected with *L. major* induces a type I transcriptional signature leading to the production of IL-12 (65). All these studies point to a common response to *Leishmania* -infection irrespective of the species. Accordingly, our study is unable to confirm the type I Interferon signaling in *Lg*-CL as our study is not ample enough to answer this finding. However, high RNA expression of CXCL10 and CXCL8 as well as high plasma levels of IFN- γ, CXCL10, CXCL8 and IL-10 were observed in PBMCs and plasma from VL patients caused by *L. donovani*, respectively (66). Another study also observed high levels of plasma IFN- γ, TNF-α, IL-6, CXCL8, and IL-10 in active VL caused by *L. infantum (chagasi)* (67). It is noteworthy to highlight that we also observed high plasma levels of IFN- γ, TNF-α, IL-6 and CXCL10, CXCL8 and IL-10 among the patients with *Lg*-CL. Spearman correlation of IFN- γ to IL-10 among the healthy controls is similar to patients with Lg-CL (ρ = 0.62). It is well-known that IL-8 attracts neutrophils at the site of infection and IFN- γ triggers macrophage activation to keep the parasites in check. Correlation of IFN- γ to IL-4 among healthy controls was also similar among HC and patients with Lg-CL (ρ = 0.7).

This study has few limitations. Comparisons of circulating biomarkers were performed between patients with *Lg*-CL and healthy individuals sharing the same endemic area. The study is a transversal investigation and analyzed the patients only before treatment. It would be interesting to identify predictive markers of treatment failure or success by comparing the immune profile in infected individuals before and after completion of treatment.

## Conclusion

Altogether, this study identifies plasma cytokines pathways that may lead to the development of disease in *Lg*-infected susceptible individuals. While common response is observed to *Leishmania*-infection, however, our study points that IP-10, IL-2 and RANTES could be a predictive value of clinical parameters for progressing to disease, while PDGF, IL-1Ra and eotaxin may be protective biomarkers. IL-17 and IL-1β also are potential predictive markers for progression to disease in *Lg*-infected individuals. Identifying potential target in these pathways may open the way for immunotherapy.

## Data availability statement

The original contributions presented in the study are included in the article/**Supplementary Material**. Further inquiries can be directed to the corresponding author.

## Ethics statement

The studies involving human participants were reviewed and approved by Research Ethics Committee of the Fundação de Medicina Tropical Doutor Heitor Vieira Dourado (FMT-HVD). Written informed consent to participate in this study was provided by the participants’ legal guardian/next of kin.

## Author contributions

TM, MS, and RR contributed for data curation. TM, JJ, MG, and RR take responsibility for the integrity of the work as a whole, from inception to published article. TM, JJ, and RR were responsible for study design and conception and drafted the manuscript. TM, JJ, LOS, GVS, FA, SP, HK, LSS, LMS, SA, KQ, JS, CS, and HS recruited healthy controls in endemic areas. TM, JJ, MG, and RR collected and cleaned the data for formal analysis. GSP and AB were responsible for statistical analysis. TM and RR interpreted the results and drafted the manuscript. All authors revised the manuscript for important intellectual content.

## Funding

This research was funded by the Conselho Nacional de Desenvolvimento Científico e Tecnológico (CNPq), grant number 404181/2012-0 to RR, Fundação de Amparo à Pesquisa do Estado do Amazonas (FAPEAM), grant number 06201954/2015 to RR and FAPEAM RESOLUÇÃO N. 002/2008, 007/2018 E 005/2019 – PRÓ-ESTADO. The funders had no role in study design, data collection and analysis, decision to publish, or preparation of the manuscript.

## Acknowledgments

We thank all the patients and healthy individuals from the endemic area who have been willing to participate.

## Conflict of interest

The authors declare that the research was conducted in the absence of any commercial or financial relationships that could be construed as a potential conflict of interest.

## Publisher’s note

All claims expressed in this article are solely those of the authors and do not necessarily represent those of their affiliated organizations, or those of the publisher, the editors and the reviewers. Any product that may be evaluated in this article, or claim that may be made by its manufacturer, is not guaranteed or endorsed by the publisher.

## Supplementary material

Supplementary Figure 1 Plasma biomarkers assessed in the total population in relation to age and sex.

Supplementary Figure 2 Plasma cytokines, chemokines profile and growth factors in patients with cutaneous leishmaniasis and healthy controls. Plasma samples from Leishmania guyanensis-infected patients (PT) and healthy individuals(CT) were assessed by Luminex. The results are expressed in pg/mL and displayed as median and interquartile range. Comparative analysis between control vs patient was done by Kruskall-Wallis test and significant differences are expressed as p < 0.05, p < 0.01, p < 0.001 and p < 0.0001). For biomarkers influenced by age, P values were adjusted for age by general linear model of the R package.
