# Supplementary figures and images for: Distinct plasma chemokines and cytokines signatures in Leishmania guyanensis-infected patients with cutaneous leishmaniasis
Source: Front Immunol. 2022 Aug 25;13:974051. doi: 10.3389/fimmu.2022.974051 (PMC9453042; doi:10.3389/fimmu.2022.974051)

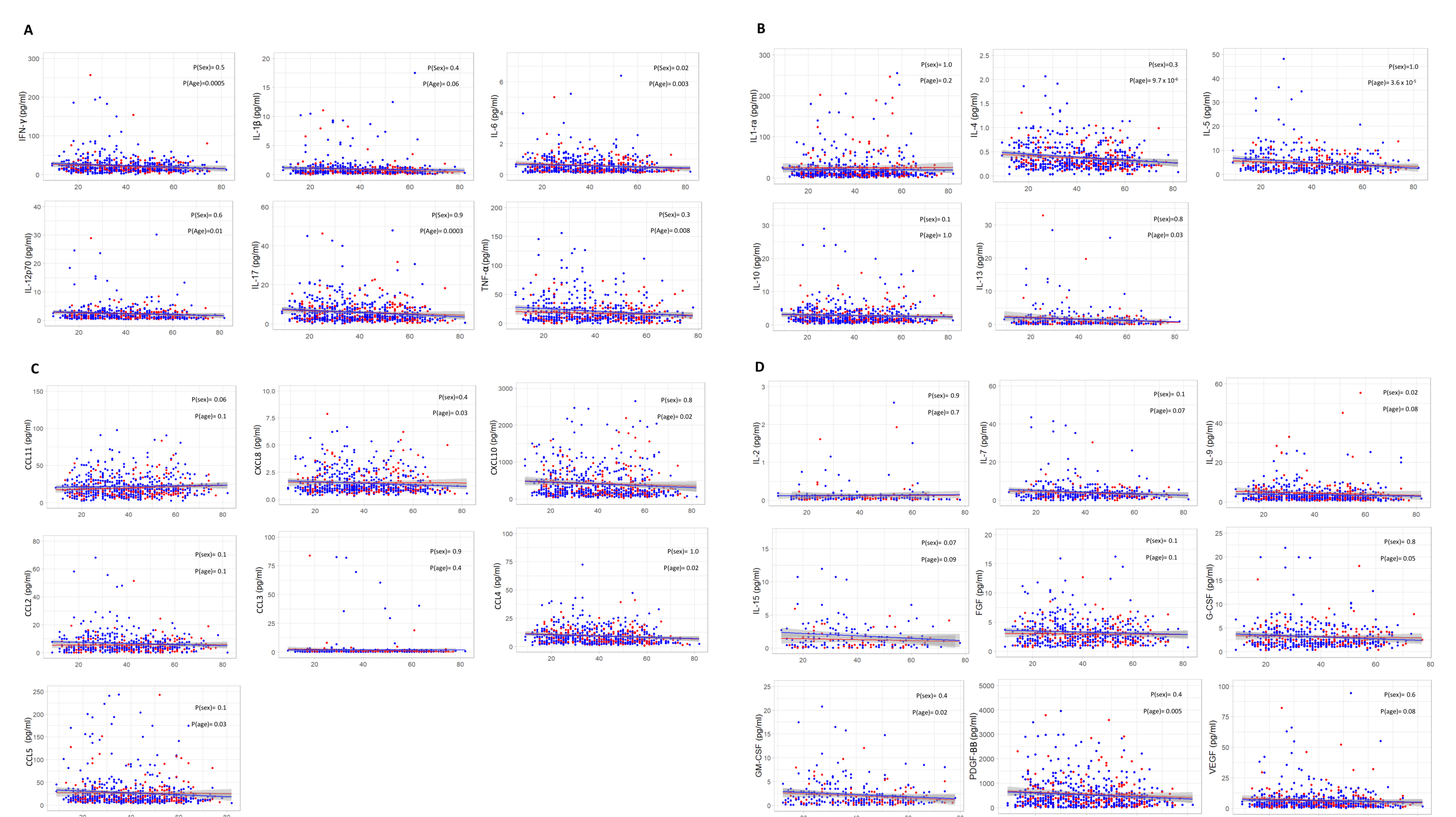

Supplement: Supplementary Figure 1 — Plasma biomarkers assessed in the total population in relation to age and sex. [file Image_1.tiff]

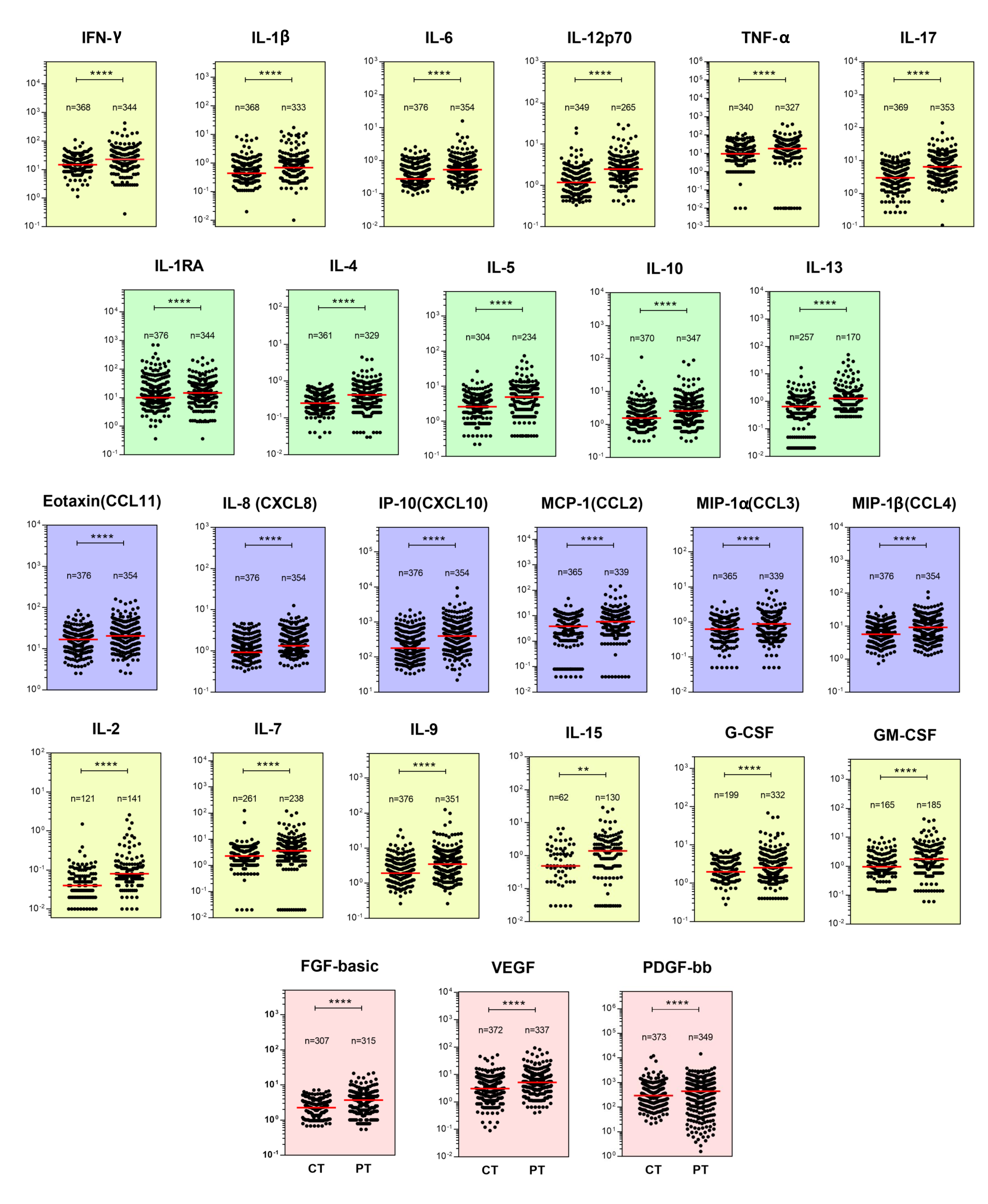

Supplement: Supplementary Figure 2 — Plasma cytokines, chemokines profile and growth factors in patients with cutaneous leishmaniasis and healthy controls. Plasma samples from Leishmania guyanensis-infected patients (PT) and healthy individuals(CT) were assessed by Luminex. The results are expressed in pg/mL and displayed as median and interquartile range. Comparative analysis between control vs patient was done by Kruskall-Wallis test and significant differences are expressed as p < 0.05, p < 0.01, p < 0.001 and p < 0.0001). For biomarkers influenced by age, P values were adjusted for age by general linear model of the R package. [file Image_2.tiff]
